# Overlapping Community Detection based on Network Decomposition

**DOI:** 10.1038/srep24115

**Published:** 2016-04-12

**Authors:** Zhuanlian Ding, Xingyi Zhang, Dengdi Sun, Bin Luo

**Affiliations:** 1 School of Computer Science and Technology, Anhui University, Hefei 230601, China; 2 Key Lab of Industrial Image Processing & Analysis of Anhui Province, Anhui Province, Hefei 230039, China

## Abstract

Community detection in complex network has become a vital step to understand the structure and dynamics of networks in various fields. However, traditional node clustering and relatively new proposed link clustering methods have inherent drawbacks to discover overlapping communities. Node clustering is inadequate to capture the pervasive overlaps, while link clustering is often criticized due to the high computational cost and ambiguous definition of communities. So, overlapping community detection is still a formidable challenge. In this work, we propose a new overlapping community detection algorithm based on network decomposition, called NDOCD. Specifically, NDOCD iteratively splits the network by removing all links in derived link communities, which are identified by utilizing node clustering technique. The network decomposition contributes to reducing the computation time and noise link elimination conduces to improving the quality of obtained communities. Besides, we employ node clustering technique rather than link similarity measure to discover link communities, thus NDOCD avoids an ambiguous definition of community and becomes less time-consuming. We test our approach on both synthetic and real-world networks. Results demonstrate the superior performance of our approach both in computation time and accuracy compared to state-of-the-art algorithms.

With the development of complex network in various fields including biological organisms and human society, community detection has become a vital step to understand the structure and dynamics of networks^1^,^2^,^3^. Although no common definition of community has been agreed upon, it is widely accepted that a community should have more internal than external connections^4^. However, many real networks have communities with pervasive overlaps^5^,^6^,^7^. For example, a person belongs to more than one social group such as family group and friend group. So, these objects should be divided into multiple groups, which are known as overlapping nodes. The aim of overlapping community detection is to discover such overlapping nodes and communities.

In the past few years, many different approaches, such as hierarchical clustering^8^, spectral clustering^9^,^10^ and optimization based algorithms^11^,^12^ have been proposed to uncover community structure in networks. These methods restrict a node to belonging to only one community and therefore result in some computational advantages. However, for real networks having complex overlapping community structures, these methods are obviously inadequate in identifying communities with overlaps^5^. For this reason, overlapping community detection has drawn lots of attention. Generally speaking, existing overlapping community detection approaches could be divided into two categories: node based algorithms (node clustering) and link based algorithms (link clustering).

The node based overlapping community detection algorithms divide nodes of the network into different communities directly, utilizing the structure information of nodes. Many well established algorithms of this type are proposed. One of such approaches is based on the idea of clique percolation theory^13^,^14^,^15^, which is the most prominent algorithm for overlapping community detection. Another type is based on local expansion or optimization^16^,^17^,^18^,^19^ among which LFM^16^, GCE^17^ and OCG^19^ are typical algorithms of this category. Besides, some fuzzy community detection algorithms calculate the possibility of each node belonging to every community, such as SSDE^20^ and IBFO^21^. However, most node based algorithms need prior information to detect overlapping communities. For example, LFM needs an appropriate parameter *α* to control the size of communities and CPM is sensitive to the parameter *k*. For fuzzy community detection algorithms, the number of communities should be determined in advance and the clustering accuracy relies on the utilized fuzzy techniques. OCG can determine the number of community automatically, while it is blamed for discovering communities with small size in some networks. Moreover, the overlap complicates the overall structure of overlapping communities to be discovered and incurs extra computation time.

To overcome the shortages of node based algorithm above, the recent studies have focused on the link based strategies. The motivation is that link communities are more intuitive than node communities in many real-world networks. According to this idea, some previous researches have shown the advantages of link community discovery in networks^22^,^23^,^24^,^25^,^26^,^27^. These algorithms are all established based on an intuition that a link usually has a unique identity and the links connected to a single node may belong to several different link communities. Specifically, Link clustering (LC) was initially proposed by Ahn *et al.*^22^ in 2010 and applied for massive networks. LC hierarchically groups the adjacent edges using an edge-shared neighborhood measure. Then, a number of followed approaches to identify link communities in networks have been proposed consecutively. For instance, Huang *et al.*^23^ propose an extended link clustering method (ELC) for overlapping community detection, with a superior performance than LC. Besides, Pan *et al.*^27^ detect link communities by a local-based method, which expands a selected seed by optimizing a proposed local function to find each natural community. These newly proposed link based algorithms seem conceptually natural and show their superiority on detecting overlapping communities. However, high computation time is cost and even there is no guarantee that it provides higher quality detection than node based algorithms do^3^, because these traditional link based algorithms always rely on an ambiguous definition of community. As an example, every link is forced into a community while there are real networks that have links that do not fit into any community, which results in typically a highly overlapping community structure. Specifically, LC emphasizes the community density and ignores the connection among communities, which could result in bias on small communities in theory. ELC may become computationally expensive in the dense network due to the complicated calculation of extended link similarity. So link community detection still poses a formidable challenge.

Hence, the study on the novel fast link clustering method can significantly speed up the discovering of overlapping communities, and facilitate the understanding of network systems. Inspired by this idea, we propose a new method for overlapping community detection on the basis of network decomposition (NDOCD). NDOCD focuses on iteratively removing links in obtained link community to split the network into smaller components and uses node clustering technique to identify link communities. Because of network decomposition and noise links elimination during optimization, both computational efficiency and the quality of obtained communities are improved. Besides, different from traditional link clustering, our link communities are obtained by employing node clustering technique rather than link similarity measure, so an ambiguous definition of community and high computational complexity are avoided. Moreover, it is unnecessary to deal with all links in the network by our method, thus reducing the computation time. Extensive experiments illustrate the competitive performance in terms of both computation time and quality of detected communities compared to state-of-the-art algorithms. Moreover, the applications on three yeast PPI networks confirm that our method is effective to predict previously unknown complexes and even unknown protein function at a low cost.

## Results

In this section, both synthetic and real-world networks are applied to test the computation time and the quality of obtained communities. The synthetic networks allow us to test the viability of different methods for known community detection under controlled conditions, while the real-world networks allow us to observe their capabilities under practical conditions. To evaluate the quality of obtained overlapping communities, we employ the widely used extended modularity (EQ)^28^ and extended normalized mutual information (ENMI)^5^,^16^ as the accuracy measures. In addition, three quality measures: *Precision*, *Recall* and *F-measure*^6^ are used to assess the quality of the predicted complexes on three yeast PPI networks derived from real-world biological data^29^,^30^,^31^.

Further, we compared the performance of NDOCD with two categories of representative approaches: node based clustering algorithms: CPM^13^ and OCG^19^, and link based clustering algorithms: LC^22^ and ELC^23^. For each algorithm, the final results were obtained after having optimized the algorithm parameters to yield the best possible results as measured by the corresponding evaluation criteria. For CPM, k ranges from 3 to 8. For LC and ELC, the threshold varies from 0.1 to 0.9 with an interval 0.1. For our method, the algorithm always performs best when threshold JS varies from 0.3 to 0.4 and threshold MD varies from 0.4 to 0.6. Note that all the experiments here are conducted on a PC with a 3.0 GHz Pentium(R) Dual-Core CPU and the Windows 7 SP1 32 bit operating system. Our programming environment is MATLAB 2010. The source code of the proposed method and the dateset and any other source files are available in Supplementary information.

Time complexity and space complexity analysis: In the phase of greedy expansion procedure, the time complexity is *O*(*ck*), where *c* is the size of local community obtained by seed expansion and *k* is the average degree of nodes in the network. Thus the time complexity of obtaining a set of communities is *O*(*c*_1_*k*_1_ + *c*_2_*k*_2_ + … + *c*_*l*_*k*_*l*_), where *l* is the number of obtained communities. Suppose *k*_*max*_ = *max*(*k*_1_, *k*_2_, …, *k*_*l*_), the overall time complexity of NDOCD is *O*(*nk*_*max*_), where *n* is the number of nodes in the network. The memory consuming of NDOCD is *O*(*m*) by sparse storage of the matrix, where *m* is the number of edges of the network.

### Synthetic networks

We empirically use the well-known LFR benchmark to test the performance of overlapping community detection methods. In the following experiments, each parameter set of LFR benchmark was generated similar to those designed by Lancichinetti *et al.*^32^. The network size *n* varies from 100 to 1000 with interval 100, the average degree *k* = 10 or *k* = 25, the maximum degree *k*_*max*_ = 50, the mixing parameter *u* varies from 0.1 to 0.6 with interval 0.1, vertex degrees and community sizes are controlled by power-law distribution with exponents *τ*_1_ = 2 and *τ*_2_ = 2 respectively, the minimum community size *c*_*min*_ = 10, the maximum community size *c*_*max*_ = 50, overlapping diversity *o*_*m*_ varies from 2 to 8, overlapping density *o*_*n*_/*n* varies from 10% to 60% with interval 10%. Here, we conducted five sets of benchmarks. The first set of LFR benchmark is used to test the computation time of different algorithms and other four benchmarks are used to evaluate the effect of the mixing parameter *u*, network size *n*, overlapping diversity *o*_*m*_ and overlapping density *o*_*n*_/*n* respectively. For each parameter set generated via LFR, we generated 10 instantiations.

**Figure 1:**
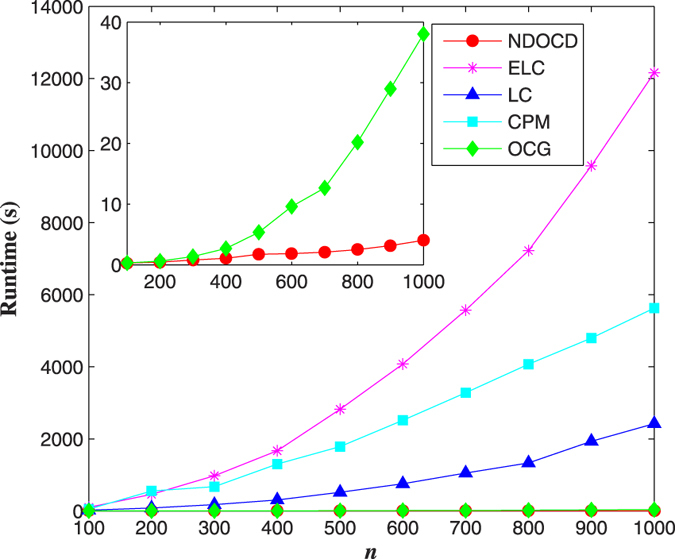
Comparison of computation time of different algorithms on synthetic networks with different sizes. Plots show runtime (s) for networks with *n* = 100 ~ 1000, *k* = 10, *k*_*max*_ = 50, *u* = 0.1, *τ*_1_ = 2, *τ*_2_ = 1, *c*_*max*_ = 50, *c*_*min*_ = 10, *o*_*m*_ = 2, *o*_*n*_/*n* = 10%.

First, we compare the computation time of different algorithms on the first set of LFR benchmarks with different network sizes. Figure 1 shows the execution time taken by the various algorithms on these considered networks. As we can see, the proposed NDOCD outperforms other four approaches and such superiority becomes significant with the increase of nodes. The main reason is attributed to the decomposition of the network and the utilized node clustering technique to discover link communities. Among all the compared algorithms, LC and ELC, two hierarchical link clustering algorithms, become computationally expensive because of complicated calculation of link similarity. CPM is time-consuming by locating maximal cliques and always fails to terminate in many large networks. OCG is an elite algorithm of high time efficiency, while NDOCD is quite competitive to OCG algorithm with runtime being even slightly better.

**Figure 2:**
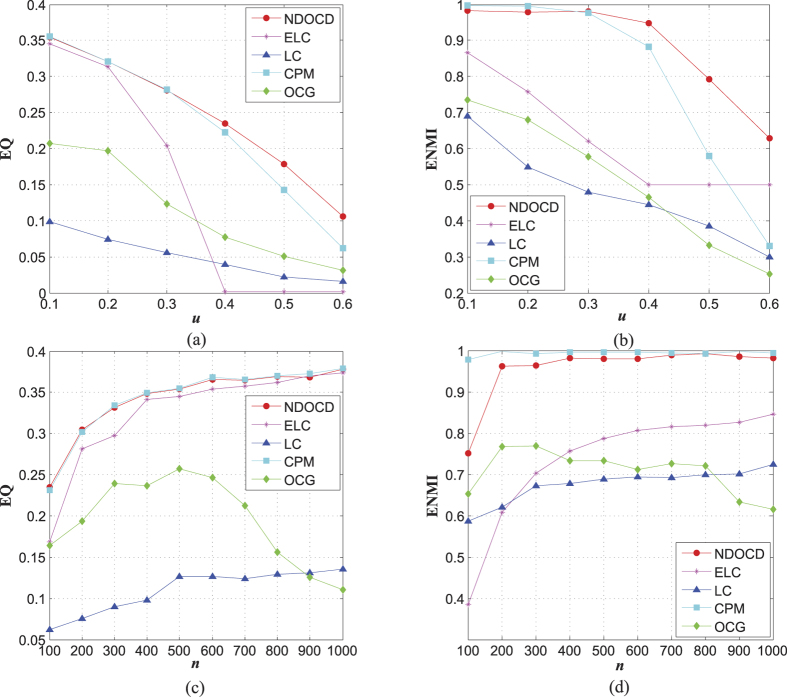
The effects of mixing parameter *u* and network size *n* on synthetic networks. (**a**) EQ for networks with *n* = 500, *k* = 25, *k*_*max*_ = 50, *u* = 0.1 ~ 0.6, *τ*_1_ = 2, *τ*_2_ = 1, *c*_*max*_ = 50, *c*_*min*_ = 10, *o*_*m*_ = 2, *o*_*n*_/*n* = 10%, (**b**) ENMI for networks with *n* = 500, *k* = 25, *k*_*max*_ = 50, *u* = 0.1 ~ 0.6, *τ*_1_ = 2, *τ*_2_ = 1, *c*_*max*_ = 50, *c*_*min*_ = 10, *o*_*m*_ = 2, *o*_*n*_/*n* = 10%, (**c**) EQ for networks with *n* = 100 ~ 1000, *k* = 25, *k*_*max*_ = 50, *u* = 0.1, *τ*_1_ = 2, *τ*_2_ = 1, *c*_*max*_ = 50, *c*_*min*_ = 10, *o*_*m*_ = 2, *o*_*n*_/*n* = 10%, (**d**) ENMI for networks with *n* = 100 ~ 1000, *k* = 25, *k*_*max*_ = 50, *u* = 0.1, *τ*_1_ = 2, *τ*_2_ = 1, *c*_*max*_ = 50, *c*_*min*_ = 10, *o*_*m*_ = 2, *o*_*n*_/*n* = 10%.

**Figure 3:**
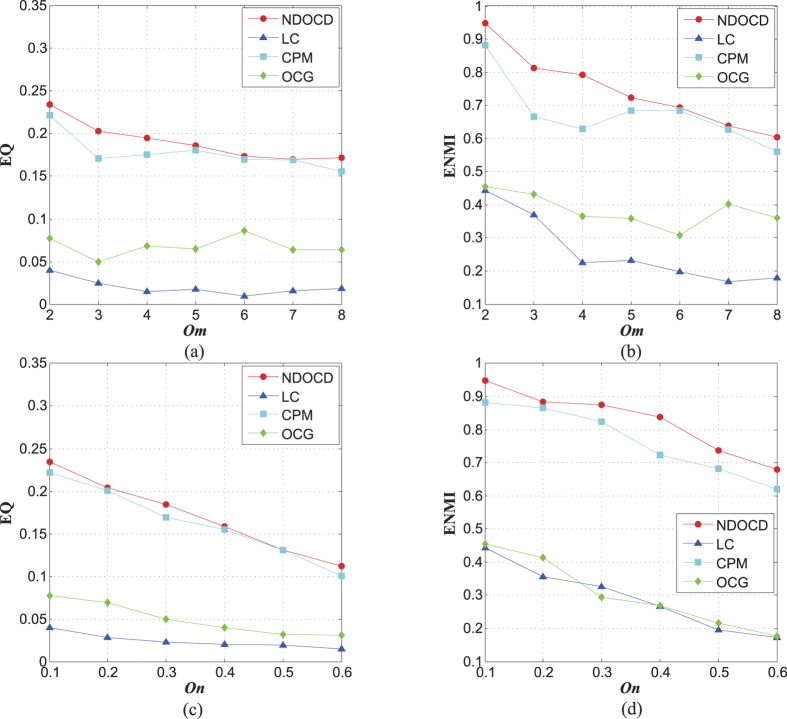
The effects of overlapping diversity *o*_*m*_ and overlapping density *o*_*n*_/*n* on synthetic networks. (**a**) EQ for networks with *n* = 500, *k* = 25, *k*_*max*_ = 50, *u* = 0.4, *τ*_1_ = 2, *τ*_2_ = 1, *c*_*max*_ = 50, *c*_*min*_ = 10, *o*_*m*_ = 2 ~ 8, *o*_*n*_/*n* = 10%, (**b**) ENMI for networks with *n* = 500, *k* = 25, *k*_*max*_ = 50, *u* = 0.4, *τ*_1_ = 2, *τ*_2_ = 1, *c*_*max*_ = 50, *c*_*min*_ = 10, *o*_*m*_ = 2 ~ 8, *o*_*n*_/*n* = 10%, (**c**) EQ for networks with *n* = 500, *k* = 25, *k*_*max*_ = 50, *u* = 0.4, *τ*_1_ = 2, *τ*_2_ = 1, *c*_*max*_ = 50, *c*_*min*_ = 10, *o*_*m*_ = 2, *o*_*n*_/*n* = 10% ~ 60%, (**d**) ENMI for networks with *n* = 500, *k* = 25, *k*_*max*_ = 50, *u* = 0.4, *τ*_1_ = 2, *τ*_2_ = 1, *c*_*max*_ = 50, *c*_*min*_ = 10, *o*_*m*_ = 2, *o*_*n*_/*n* = 10% ~ 60%.

Next, we compare the quality of obtained communities of different algorithms in terms of EQ and ENMI on the other four sets of LFR benchmarks. The performance is shown in Figs 2 and 3.

Figure 2a,b present how the performance changes on the second set of synthetic networks with different mixing parameter *u*. In general, increasing *u* typically results in poorer performance for all methods, due to the fact that all communities are mixed together and each single community is polluted by the noise links from the neighboring communities. From Fig. 2a,b, we can see that NDOCD outperforms other methods for the networks without obvious community structure (high *u*) and the gaps between our approach and other methods are more prominent in high mixing parameter *u* case. What is worse, except for NDOCD, most methods fail to deal with the networks with *u* over 0.3. In the case with high mixing parameter *u*, the performance of comparable CPM method may be compromised for these networks with weak clique presence, because many nodes are left out.

Performance for the third set of synthetic networks is summarized in Fig. 2c,d to check the effects of network size *n*. From Fig. 2c,d we conclude that increasing network size typically results in slightly better performance. Besides, for the networks with low *u*, both NDOCD and CPM get larger EQ and ENMI than their counterparts, but NDOCD can not perform as better as in the case without obvious community structure. Among all the compared algorithms, CPM performs best in this case. However, the performance of CPM drops significantly with high *u* shown in Fig. 2a,b.

The remaining two sets of synthetic networks are used to evaluate the effects of overlapping diversity *o*_*m*_ and overlapping density *o*_*n*_/*n* in high *u* case respectively, since high *u* brings networks with weak community structure closer to the features observed in real-world networks. Notice that ELC fails to detect the communities here due to the fact that ELC finds merely one single giant community in these benchmarks with *u* over 0.3, so we ignore ELC in our comparison here.

We first examine how the performance changes as overlapping diversity *o*_*m*_ varies from small to large values in Fig. 3a,b, and then verify the effect of overlapping density *o*_*n*_/*n* in Fig. 3c,d. From Fig. 3, we can conclude that detection performance of all algorithms consistently drops both in high overlapping diversity case and high overlapping density case. In these benchmarks, both NDOCD and CPM show competitive performance while NDOCD outperforms CPM slightly both in EQ and ENMI. Notice that LC and OCG algorithms show their weakness here, this is because they often find the significant numbers of small communities and fail to detect the communities defined in these benchmarks.

**Figure 4:**
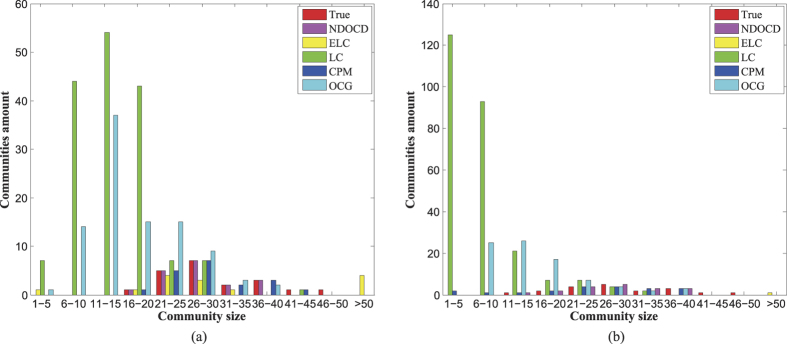
Histogram of the detected community sizes on LFR benchmark. (**a**) Comparison on networks with *n* = 500, *k* = 25, *k*_*max*_ = 50, *u* = 0.1, *τ*_1_ = 2, *τ*_2_ = 1, *c*_*max*_ = 50, *c*_*min*_ = 10, *o*_*m*_ = 2, *o*_*n*_/*n* = 10%, (**b**) Comparison on networks with *n* = 500, *k* = 25, *k*_*max*_ = 50, *u* = 0.4, *τ*_1_ = 2, *τ*_2_ = 1, *c*_*max*_ = 50, *c*_*min*_ = 10, *o*_*m*_ = 2, *o*_*n*_/*n* = 10%.

Finally, we analyze the detected community size distribution on LFR to further insight into the behaviors of different algorithms and compare it with the known ground truth. Here we only present analysis for two cases. One is the network with obvious community structure shown in Fig. 4a and the other one without obvious community structure shown in Fig. 4b. As shown in Fig. 4, both in two cases, NDOCD and CPM find communities whose sizes are distributed in agreement with the ground truth distribution, especially for NDOCD. This explains why they perform well with respect to ranking EQ and ENMI as shown in the above Fig. 2. For LC and OCG algorithms, such a distribution creates relatively significant numbers of small communities and lowers their performance. Here, we conclude that observations on the community size distribution can be used to verify the ranking and explain the performance.

### Real-world networks

In this subsection, we first test runtime(s) and clustering quality on nine real networks, including Karate network^33^, Dolphin network^34^, Football network^4^, Jazz network^35^, Metabolic network^36^, Email network^37^, PPI-D1^6^,^38^, PPI-D2^6^ and Y2H (yeast two-hybrid)^6^,^23^ listed in Table 1. Table 2 illustrates runtime(s) and EQ of all methods. Given that the ground truth is not available for most of these networks, we select EQ as the quality metric. From Table 2 we can get the following two observations. One is that NDOCD performs better in terms of runtime compared to other algorithms, and such superiority becomes more significant when the network becomes larger. In general, CPM has satisfying time efficiency in networks with highly sparse structure such as Karate and Y2H, however, the performance significantly drops for dense networks as clique detection is very time-consuming in this case. Consequently, CPM fails to deal with Jazz network of which average degree is 27.697. Besides, LC and ELC hold the same weakness for dense networks as link similarity calculation is quite time consuming. That is, the denser the network, the poorer time efficiency. OCG is a competitive fast algorithm. However, merging process becomes time consuming when there exist large number of initial clusters.

The other observation is that NDOCD outperforms the other four algorithms in terms of EQ. This confirms that for real-world networks with complicated organizational structures, our method exhibited even better relative performance to all the other methods. The observation is in agreement with the fact that our algorithm can achieve better performance on networks without obvious community structure as shown in Fig. 2a,b. Therefore, we can conclude that the proposed NDOCD is a new effective approach particularly suitable for detecting complex overlapping community structures.

Next, we examine algorithm performance on a high school friendship network where the ground truth is a total of 6 communities, shown in Supplementary Fig. S1. Even though there are no overlapping nodes reported by the students, each algorithm reports some by its own. We also include EQ, ENMI and the number of communities for reference. Results are presented in Table 3. As shown in Table 3, our approach achieve higher EQ and ENMI compared to others, so our method proves superior performance in this social network. From Table 3 we observe that some algorithms tend to over-detect the overlap and over-detect the communities, especially for LC and OCG methods, resulting in low performance in this instance. Besides, It is easy to verify that the overlapping nodes detected by our method, i.e. nodes 32, 46, 62, lie between different communities with strong connections to each individual one. Moreover, nodes 46 and 62 are also multiclustered by CPM, LC and OCG algorithms, so these nodes are the most likely to be considered as “overlapping”.

**Figure 5:**
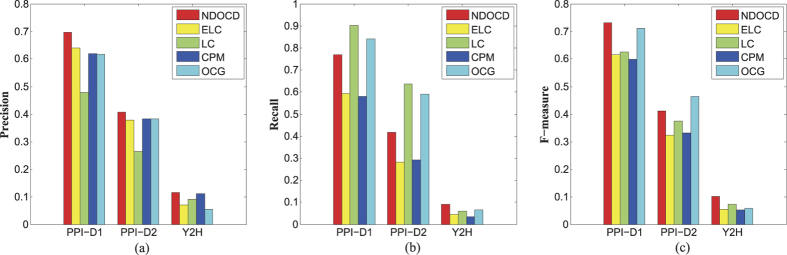
*Precision*, *Recall* and *F*-*measure* values for PPI-D1, PPI-D2 and Y2H. (**a**) *Precision* values for PPI-D1, PPI-D2 and Y2H, (**b**) *Recall* values for PPI-D1, PPI-D2 and Y2H, (**c**) *F*-*measure* values for PPI-D1, PPI-D2 and Y2H.

Furthermore, we apply our NDOCD algorithm for protein complex detection on three different yeast PPI networks, i.e. PPI-D1, PPI-D2 and Y2H listed in Table 1. We use Cmplx1 for PPI-D1, Cmplx2 for PPI-D2 and Cmplx3 for Y2H as reference sets of gold standard complexes. Cmplx1 comprises of 81 complexes of sizes at least 5 created from MIPS^39^. Cmplx2 includes 162 hand-curated complexes of sizes no less than 4 derived from MIPS^40^. Finally, Cmplx3 (mips_3_100) is created from the MIPS golden standards^41^. Figure 5 presents the *Precision*, *Recall* and *F*-*measure* values for all methods. From Fig. 5a we observe that our method obtains higher *Precision* values compared with other four methods on all the considered datasets. This is because the fact that NDOCD can find communities of reasonable size involving many reference complexes. Higher *Precision* means that a more accurate prediction, due to the predicted complexes are composed by a high percentage of proteins belonging to the reference complexes, thus the fraction of false positive is low. However, experiments reveal an imbalance in *Precision* and *Recall* for some algorithms. In this case, as shown in Fig. 5b, *Recall* of NDOCD is superior to all the other approaches on Y2H, while LC and OCG overcome NDOCD on PPI-D1 and PPI-D2. LC and OCG obtain better value of *Recall* mainly due to the fact that they find significant numbers of communities. Regarding *F*-*measure*, it is a cumulative measure considering both *Precision* and *Recall*. A high value of *F*-*measure* means that both *Precision* and *Recall* are sufficiently high. As shown in Fig. 5c, NDOCD achieves the best value of *F*-*measure* on PPI-D1 and Y2H, while on PPI-D2 NDOCD performs the second best value of *F*-*measure* among all the compared algorithms. Overall, the proposed NDOCD is quite suitable for overlapping protein complexes detection in protein-protein interaction networks.

**Figure 6:**
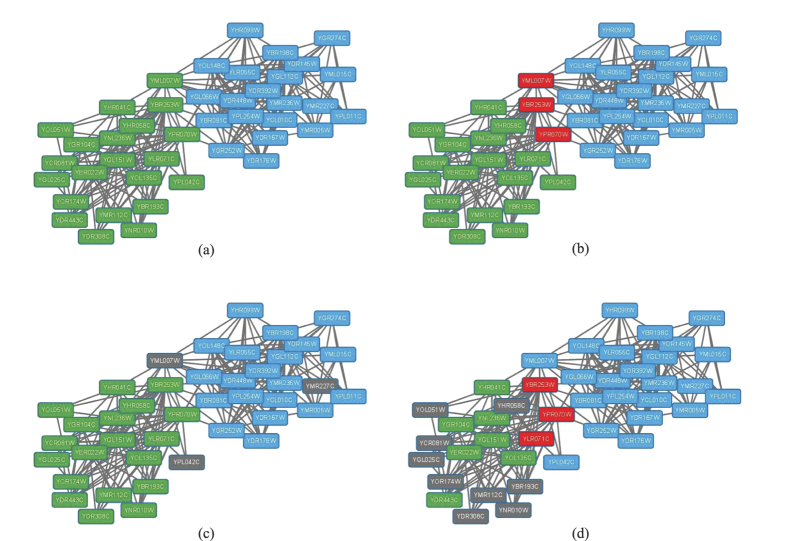
Visualization of reference and predicted complexes in PPI-D1 for NDOCD, LC and OCG. (**a**) Visualization of reference complexes, (**b**) Visualization of predicted complexes for NDOCD, (**c**) Visualization of predicted complexes for LC, (**d**) Visualization of predicted complexes for OCG.

We further visualize overlapping protein complexes in PPI networks detected by all overlapping clustering algorithms. Here, we present an example of two reference complexes labeled as #29 (blue) and #40 (green) respectively in PPI-D1 and the corresponding predicted complexes for all approaches in Fig. 6. Red nodes denote overlapping proteins belong to both complexes and grey nodes represent undiscovered proteins in complexes. Notice that CPM and ELC fail to detect the reference complexes, so no visualization exists. Two reference complexes are shown in Fig. 6a and they are both discovered correctly by our NDOCD algorithm. Moreover, three overlapping proteins YBR253W, YML007W and YPR070W are revealed in NDOCD and YML007W and YPR070W are also multiclustered by OCG method shown in Fig. 6b. So, these two multiclustered proteins are the most likely candidates for multifunctionality. From Fig. 6c,d, we can conclude that many proteins can not be detected in complexes results in a low clustering *Precision* for LC and OCG, verified in Fig. 5a.

## Discussion

In this paper, we propose a novel method for overlapping community detection from the network decomposition perspective on the basis of alternating node partition and link partition. NDOCD employs node clustering technique to identify link communities and iteratively removes all links in obtained link community to split the network into smaller components. The network decomposition and the utilized node clustering technique mainly contribute to making the algorithm more efficient and less time-consuming.

We have assessed our NDOCD method on both synthetic and real-world networks. Compared with the state-of-the-art overlapping community detection methods, experimental results show the superior performance both on time and accuracy of our method. Our NDOCD provides elegant solutions for overlapping community detection, especially for the network with complicated structures or certain amount of noise links. Moreover, we apply our approach to predict protein complexes in yeast PPI networks. Our results suggest that the proposed method is likely to identify previously unknown complexes and predict unknown protein function at a much lower cost, which is of great significance. In addition, the proposed method also can be easily applied to many other important tasks in bioinformatics, for example DNA binding protein analysis^42^, the relationship between microRNAs and disease^43^,^44^,^45^,^46^, etc. These problems will be further studied.

Departure from the existing overlapping community detection methods, our method accommodates the coexistence of node and link communities beyond the existing work for finding node or link communities separately. We employed a different way, called node clustering technique, to identify link communities. Compared with other partitioning schemes, such as node clustering methods that focus mainly on nonoverlapping communities and link clustering methods that typically produce highly overlapping communities, the new scheme can better describe the natural community structures of complex networks. Specifically, we design a novel node clustering technique which is more appropriate for our algorithm framework, rather than employing the tranditional node clustering techniques as the local optimization procedure. As we known, the quality of network decompostion influences directly the subsequent optimization. Here, to minimize the effects, the centred clique is treated as the seed to ensure the accuracy and the speed of local community, considering both joint strength and membership as the expansion rule simultaneously. While some traditional methods, including CPM and OCG, concentrate on the merging strategy for some relatively smaller components, which can not detect the natural local communities directly. Furthermore, some other strategies, such as LFM method, depend frequently on the performance of designed expansion criterion function. Apparently, the accuracy of network partition may be discounted, that is why we propose a novel note clustering method to capture better local commnities in our proposed framework. It is noteworthy that there are two parameters in our node expansion rule, and we need to adjust them to obtain the good results. Such reason makes us to design a more reasonable nonparameter node clustering technique, which we leave for future work.

Recently, several community detection methods on combining structure and content have already been proposed for the networks with a lot of content on nodes and links. Needless to say, the community detection may be greatly improved by considering both the network topology and node/link content, especially for the network with complicated structures or some noise, but this seems to be a challenge. So incorporating node and link content into our approach to even more accurately identify the overlapping communities is the subject of our future work. Also, some bio-inspired computing models and framework, for example, neural networks^47^,^48^,^49^,^50^,^51^,^52^,^53^, membrane computing^54^,^55^,^56^, virus machines^57^ and evolutionary computation^58^,^59^, might bring some ideas to improve the proposed method.

Finally, as shown in a series of recent publications^60^,^61^,^62^, user-friendly and publicly accessible web-servers can significantly enhance their impacts, we shall make efforts in our future work to provide a web-server to displaying findings that can be manipulated by users according to their need.

## Methods

In this section, we first depict the network decomposition procedure using a simple example to show the fundamental idea of our method; then we specify the overview of NDOCD; and finally we present the other core concepts of NDOCD, including seed selection and seed expansion.

### Network decomposition

**Figure 7:**
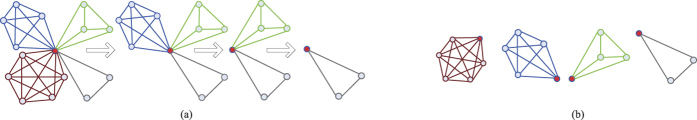
An illustration of our main idea. (**a**) Network decomposition procedure of our method, (**b**) Result of our method.

Figure 7a presents an input network and the network decomposition procedure of NDOCD for this network. Firstly, as orange link community is detected, all links in orange link community are removed from the input network. After deleting these links, the remaining network’s topology structure will appear to be simplified. By doing this repeatedly, we obtain the following sub-networks successively. Finally, all the detected non-overlapping link communities naturally determine the final division results for the nodes in the network with corresponding node communities that can be overlapped. As shown in Fig. 7, four link communities have common connected node (the red node) in the original network. As expected, the result shown in Fig. 7b, match the ground-truth given in Fig. 7a.

**Figure 8:**
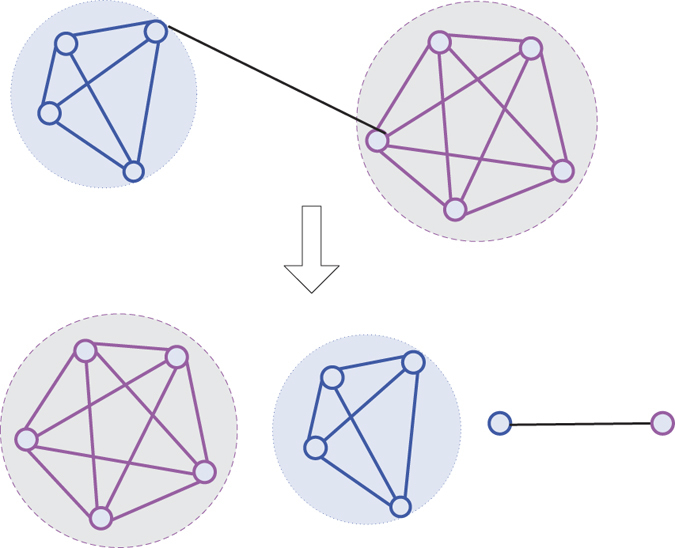
An example of bridge edge in a network. The black line represents the bridge edge in the network.

From this example we can conclude that NDOCD is a promising overlapping community detection algorithm with the following advantages, which outperforms traditional link clustering and node clustering algorithms. First, the decomposition of network contributes to reducing the computation time of NDOCD. Second, our method does not force every link into a community (all links but the bridge edge) shown in Fig. 8, thus avoid the problem that traditional link clustering typically generates a highly overlapping community structure. In addition, using node clustering method to get link communities can also ensure the quality of clustering.

### Overview of NDOCD

The detailed steps of our NDOCD algorithm are described as follows:

- Step 1: Seed selection. Identify the centred clique as starting seed by a greedy polynomial algorithm.
- Step 2: Seed expansion. Expand a single seed by local optimization strategy.
- Step 3: Network decomposition. Remove all links in derived link communities from current network.
- Step 4: Continue to loop back to step 1 until no seeds can be found.
- Step 5: Eliminate nodes with bad contribution to extended modularity of the communities.

Our algorithm consists of three major steps. The core step is the decomposition procedure described above. We iteratively remove all links in derived link communities from current network. In our method, a node community is obtained by seed expansion and all links in this node community create the corresponding link community. NDOCD utilizes node clustering technique to discover link communities, thus avoid the time-consuming link similarity calculation of traditional link clustering, especially for dense networks. The other two important steps are the following seed selection and seed expansion.

### Seed selection

We utilize cliques as seeds, which is motivated by the observation that cliques are one of the characteristic structures contained within communities. As clique detection in a graph is generally computationally expensive, we employ the centred cliques^19^, which are built using a greedy polynomial algorithm to form seeds. The resulting centred clique is not necessarily the maximal clique. Centered clique is calculated as follows:

- Step 1: Select a single vertex *x* with highest comprehensive network feature value (CNFV, defined as formula 1).
- Step 2: Build the clique centered in *x*. If a clique is produced, vertices adjacent to *x* are added in decreasing order of their relative degree.

The comprehensive network feature of node *i* reveals the joint strength between this node and other nodes in the network and the CNFV^21^ of node *i* is defined as follows:


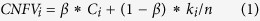


where *C*_*i*_ is the clustering coefficient of node *i* and *k*_*i*_ is the degree of node *i*, and *n* is network size. Ref. 21 shows the optimal value of parameter *β* is 0.3.

### Seed expansion

Assume that the obtained centred clique *S* is starting seed, which is identified as the core of community *C*. In general, *S* is embedded in some larger community *C*. Thus, our task is to expand the seed S by greedy local optimization. Specifically, we expand the core by adding the neighbor nodes whose joint strength (*JS*, defined as formula 2) or membership degree (*MD*, defined as formula 3) reaches the specified thresholds until all nodes do not satisfy the condition.

The *JS* of node *i* to graph *K* is


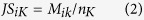


The *MD* of node *i* to graph *K* is


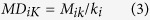


where *M*_*ik*_ is the total links between node *i* and graph *K*, and *n*_*K*_ is total nodes in graph *K*.

Finally, a filtering process is added. Eliminate loosely assigned nodes with a threshold within 0 and 1 according to contribution of each node to the extended modularity of the communities and discard communities that contain less than two nodes, thus further improving the quality of obtained communities.

## Additional Information

**How to cite this article**: Ding, Z. *et al.* Overlapping Community Detection based on Network Decomposition. *Sci. Rep.* **6**, 24115; doi: 10.1038/srep24115 (2016).

## Supplementary Material

Supplementary Information

## Figures and Tables

**Table 1:** Real-world networks used in the experiments.

Networks	Nodes	Edges	Averagedegree	Description
Karate	34	78	4.588	Zachary’s karate club
Dolphins	62	159	5.129	Dolphins social network
Football	115	613	10.661	American college football
Jazz	198	2742	27.697	Jazz musicians network
Metabolic	453	2025	8.940	C. elegans metabolic network
Email	1133	5451	9.622	Email network URV
PPI-D1	990	4687	9.469	Yeast PPI dataset1
PPI-D2	1443	6993	9.692	Yeast PPI dataset2
Y2H	2018	2930	2.904	Yeast two hybird

**Table 2:** Experimental results on nine real-world networks.

Network	Runtime(s)					*EQ*				
	LC	ELC	CPM	OCG	NDOCD	LC	ELC	CPM	OCG	NDOCD
Karate	0.61	2.49	0.67	0.23	**0.20**	0.1448	0.1633	0.1147	0.0855	**0.2055**
Dolphins	1.97	8.96	2.00	0.25	**0.20**	0.1368	0.1920	0.1870	0.1196	**0.2392**
Football	25.22	141.11	28.67	0.37	**0.31**	0.1762	0.1956	**0.2839**	0.2691	0.2746
Jazz	691.94	3087.00	–	1.11	**0.33**	0.0332	0.1301	0.1133	0.0322	**0.1873**
Metabolic	392.44	1655.57	554.76	9.07	**0.66**	0.0509	0.0679	0.0494	0.0674	**0.0951**
Email	3785.91	15228.46	1580.07	107.71	**13.68**	0.0585	0.1714	0.1327	0.0638	**0.1896**
PPI-D1	2085.80	8843.52	10771.28	33.71	**2.58**	0.1604	0.3590	0.2049	0.1703	**0.3620**
PPI-D2	4590.79	22048.35	102347.28	96.89	**6.40**	0.1310	0.3552	0.2217	0.1314	**0.3672**
Y2H	637.40	3917.59	56.22	360.73	**11.95**	0.1157	0.2256	0.0578	0.1201	**0.2221**

In the table, the dash denotes run time over 72 hours.

**Table 3:** Test on a high school friendship network.

Algorithm	Communitynumber	Overlapping nodes	EQ	ENMI
LC	15	total 26	0.1507	0.4422
ELC	4	{1, 13, 19, 32, 49, 59, 67}	0.2556	0.4065
CPM	7	{19, 46, 47, 50, 62}	0.2189	0.3392
OCG	29	total 40	0.1045	0.3750
NDOCD	5	{32, 46, 62}	**0.2984**	**0.6741**

For algorithms that discover more than 10 overlapping nodes, only the total number is shown.
