# Supplementary material for: Overlapping Community Detection based on Network Decomposition
Source: Sci Rep. 2016 Apr 12;6:24115. doi: 10.1038/srep24115 (PMC4828636; doi:10.1038/srep24115)
Supplement: Supplementary Information [file srep24115-s1.pdf]

Supplementary Materials

Overlapping Community Detection based on Network Decomposition

Zhuanlian Ding, Xingyi Zhang, Dengdi Sun,* and Bin Luo

Contents

Supplementary Figure 1

Supplementary Data... 2

Supplementary Code 3

Supplementary Figure

Supplementary Fig. S1.

High school friendship network (n = 69, k = 6.4). Colors represent known communities corresponding to 6 different grades.

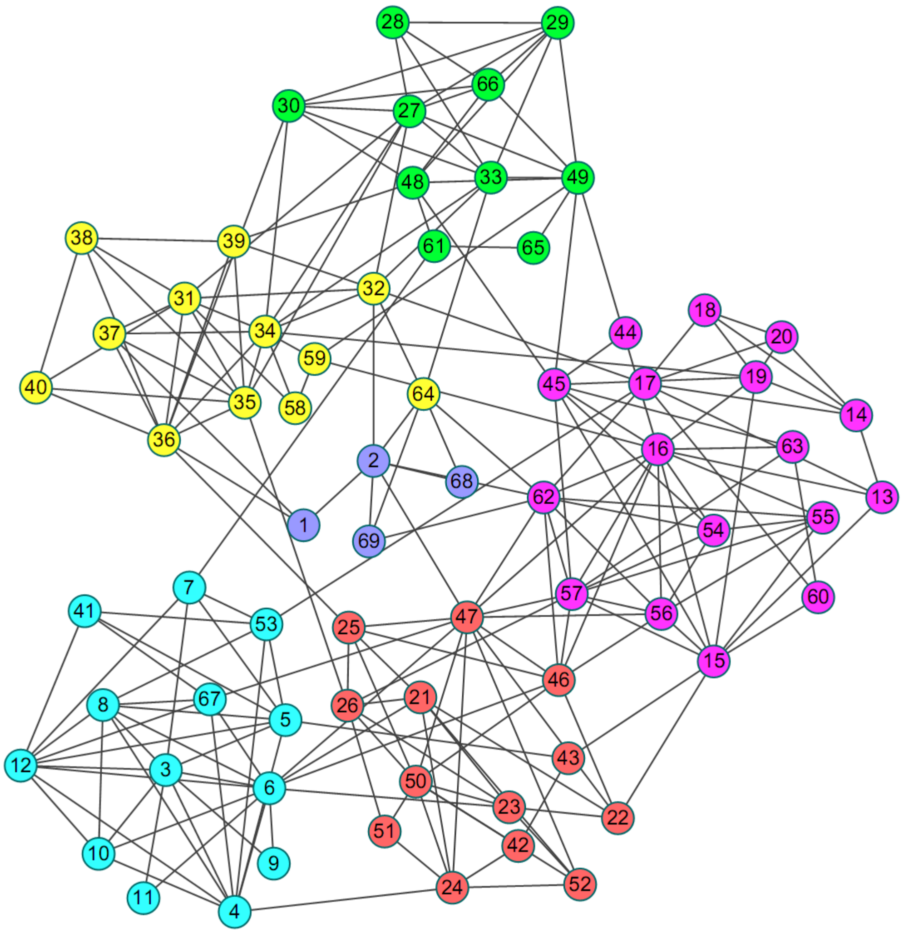

Supplementary Data

In this paper, we perform the experiments on ten real networks, including

Karate network (file name: karate.txt),

Dolphin network (filename: dolphins.txt),

Football network (filename: football.txt),

Jazz network (filename: jazz.txt),

Metabolic network (filename: metabolic.txt),

Email network (filename: emal.txt),

Highschool network (filename: highschool.txt),

PPI-D1 (filename: PPI-D1.txt),

PPI-D2 (filename: PPI-D2.txt),

Y2H (filename: Y2H-union.txt).

Here, we use Cmplx1 for PPI-D1, Cmplx2 for PPI-D2 and Cmplx3(mips_3_100) for Y2H as reference sets of gold standard complexes. The correspoing files are Cmplx-D1.txt, Cmplx-D2.txt and mips_3_100.txt, respectively. Also, we have the groundtruth of communities of Highschool network, and the file is highschoolTrue_Cl.mat.

In addtion, we empirically use the well-known LFR benchmark to test the performance of overlapping community detection methods. The network generator tool is in the file "LFR generator".

Supplementary Code

The source code, including our method (NDOCD) and three comparison methods (CPM, LC & ELC, and OCG).
